# Case Report: ANCA-Associated Vasculitis Presenting With Rhabdomyolysis and Pauci-Immune Crescentic Glomerulonephritis After Pfizer-BioNTech COVID-19 mRNA Vaccination

**DOI:** 10.3389/fimmu.2021.762006

**Published:** 2021-09-30

**Authors:** Samy Hakroush, Björn Tampe

**Affiliations:** ^1^ Institute of Pathology, University Medical Center Göttingen, Göttingen, Germany; ^2^ Department of Nephrology and Rheumatology, University Medical Center Göttingen, Göttingen, Germany

**Keywords:** coronavirus disease 2019 (COVID-19), vaccination, anti-neutrophil cytoplasmic antibody (ANCA), ANCA-associated vasculitis (AAV), rhabdomyolysis, acute kidney injury (AKI), pauci-immune crescentic glomerulonephritis (GN)

## Abstract

As the coronavirus disease 2019 (COVID-19) pandemic is ongoing and new variants of severe acute respiratory syndrome coronavirus type 2 (SARS-CoV-2) are emerging, there is an urgent need for COVID-19 vaccines to control disease outbreaks by herd immunity. Surveillance of rare safety issues related to these vaccines is progressing, since more granular data emerge with regard to adverse events of COVID-19 vaccines during post-marketing surveillance. Interestingly, four cases of anti-neutrophil cytoplasmic antibody (ANCA)-associated vasculitis (AAV) presenting with pauci-immune crescentic glomerulonephritis (GN) after COVID-19 mRNA vaccination have already been reported. We here expand our current knowledge of this rare but important association and report a case of AAV presenting with massive rhabdomyolysis and pauci-immune crescentic GN after Pfizer-BioNTech COVID-19 mRNA vaccination. As huge vaccination programs are ongoing worldwide, post-marketing surveillance systems must continue to assess vaccine safety important for the detection of any events associated with COVID-19 vaccination. This is especially relevant in complex diseases where diagnosis is often challenging, as in our patient with AAV presenting with massive rhabdomyolysis and pauci-immune crescentic GN.

## Introduction

As the coronavirus disease 2019 (COVID-19) pandemic is ongoing and new variants of severe acute respiratory syndrome coronavirus type 2 (SARS-CoV-2) are emerging, there is an urgent need for COVID-19 vaccines to control disease outbreaks by herd immunity (1). The use of novel vaccines containing a nucleoside-modified messenger ribonucleic acid (mRNA) or a viral deoxyribonucleic acid (DNA) vector that encodes the viral spike (S) glycoprotein of SARS-CoV-2 has already been approved. Large clinical trials have shown that these COVID-19 vaccines are safe and effective. Common adverse events include mild to moderate reactions at the injection site, fever, fatigue, body aches, and headache (2). Surveillance of rare safety issues related to these vaccines is progressing, since more granular data emerge with regard to adverse events of COVID-19 vaccines during post-marketing surveillance (3). Anti-neutrophil cytoplasmic antibody (ANCA)-associated vasculitis (AAV) is a small vessel vasculitis hallmarked by the presence of antibodies against autoantigens in cytoplasmic granules of neutrophils (4). AAV presents as granulomatosis with polyangiitis (GPA), microscopic polyangiitis (MPA), and eosinophilic granulomatosis with polyangiitis (EGPA) (5). Generally, renal manifestations in AAV are estimated at 80% among all cases mainly manifesting as ANCA-associated glomerulonephritis (ANCA GN), and the overall prevalence does not seem to differ substantially between MPO-ANCA and PR3-ANCA AAV (6). Interestingly, five cases of renal AAV presenting with pauci-immune crescentic ANCA GN after COVID-19 mRNA vaccination have already been reported (7–10). We here expand our current knowledge of this rare but important association and report a case of AAV presenting with massive rhabdomyolysis and pauci-immune crescentic GN after Pfizer-BioNTech COVID-19 mRNA vaccination.

## Case Report

**Figure 1:**
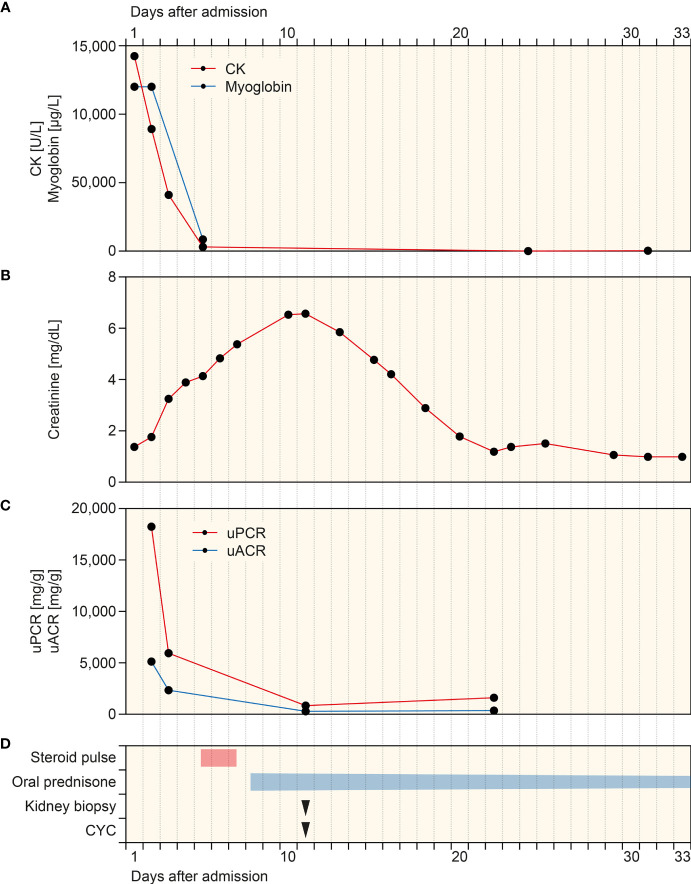
Timeline of the case after admission. **(A–C)** Time course of CK, myoglobin, plasma creatinine, and levels of uPCR and uACR. **(D)** Time of treatment regimens and kidney biopsy. CK, creatinine kinase; CYC, cyclophosphamide; uACR, urinary albumin-to-creatinine ratio; uPCR, urinary protein-to-creatinine ratio.

A 79-year-old Caucasian female with a past medical history of hypertension, degenerative disc disease, and no documented history of COVID-19 received two doses of Pfizer-BioNTech COVID-19 mRNA vaccination. Two weeks thereafter, the patient presented to our emergency department with weakness and upper thigh pain. Vital parameters were stable, and physical examination was unremarkable. The patient had no allergies and denied illicit drug use. External routine laboratory assessments obtained 1 week prior to admission were normal for serum creatinine of 0.71 mg/dl (reference range: 0.5–0.95), estimated glomerular filtration rate (eGFR) of 84.4 ml/min/1.73 m^2^, and urinalysis with the absence of hematuria or proteinuria. Repeat reverse transcription polymerase chain reaction (RT-PCR) testing for SARS-CoV-2 RNA from nasopharyngeal swabs was negative. Laboratory assessments at admission showed massive rhabdomyolysis with creatinine kinase (CK) levels of 14,243 U/L (reference range: 29–168), myoglobinemia of >12,000 µg/L (reference range: ≤106, **Figure 1A**), and acute kidney injury (AKI) with serum creatinine levels of 1.38 mg/dl (reference range: 0.7–1.2, **Figure 1B**) and an estimated glomerular filtration rate (eGFR) of 33.5 ml/min/1.73 m^2^. Urinary analysis revealed leukocyturia, hematuria (no dysmorphic erythrocytes), few renal tubular epithelial cells, and nephrotic range proteinuria of >18,000 mg/g creatinine and albuminuria of <5,000 mg/g creatinine (reference range: <30 mg/g, **Figure 1C**). The patient received intravenous crystalloids with decreasing CK levels and myoglobinemia (**Figure 1A**). However, progressive deterioration of kidney function with worsening of serum creatinine levels up to 6.57 mg/dl (reference range: 0.7–1.2 mg/dl, **Figure 1B**) and an eGFR of <15 ml/min/1.73 m^2^ occurred. ANCA immunofluorescence (IF) was positive at 1:1,000 (reference range: <1:10) with elevated MPO-ANCA levels >134 IU/ml (reference range: <3.5 IU/ml), while myositis antibodies, complement levels, and other serologic parameters were all tested negative (**Table 1**). Because of leukocytosis, a white blood differential was conducted revealing prominent peripheral blood eosinophilia (**Table 1**).

**Table 1:** Serologic parameters after admission.

	Value	Reference range
Serologic parameters		
HIV Ag/Ab—titer	Neg	Neg
HBsAg—titer	Neg	Neg
Anti-HCV—titer	Neg	Neg
Rheumatoid factor—IU/ml	<10	<15.9
Complement C3c—g/L	0.97	0.82–1.93
Complement C4—g/L	0.19	0.15–0.57
ANCA IF	1:1,000	<1:10
PR3-ANCA—IU/ml	<0.2	<2
MPO-ANCA—IU/ml	>134	<3.5
ENA screen	<0.1	<0.7
Anti-DFS70—U/ml	<0.6	<7
Anti-ds-DNA—IU/ml	4.4	<15
Histones—U/ml	7.7	<20
ANA IF	1:320	<1:100
RO52—blot	Neg	Neg
PM-Scl-100—blot	Neg	Neg
PM-Scl-75—blot	Neg	Neg
Ku—blot	Neg	Neg
SRP—blot	Neg	Neg
PL-7—blot	Neg	Neg
PL-12—blot	Neg	Neg
EJ—blot	Neg	Neg
OJ—blot	Neg	Neg
JO1—blot	Neg	Neg
Mi alpha—blot	Neg	Neg
Mi-2 beta—blot	Neg	Neg
TIF1 gamma—blot	Neg	Neg
MDA-5—blot	Neg	Neg
NXP2—blot	Neg	Neg
SAE1—blot	Neg	Neg
White blood differential		
Leukocytes—1,000/µl	22.9	4–11
Lymphocytes—%	4.7	20–45
Monocytes—%	4.5	3–13
Eosinophils—%	23.3	≤8
Basophils—%	0.2	≤2
Neutrophils—%	67.3	40-76

ANA, antinuclear antibodies; ANCA, anti-neutrophil cytoplasmic antibody; ds-DNA, double stranded-DNA; DSF70, dense-fine-speckled 70; ENA, EJ, glycine; ENA, extractable nuclear antigen; HBsAg, hepatitis B surface antigen; HCV, hepatitis C virus; HIV, human immunodeficiency virus; JO1, histidyl tRNA synthetase; MDA-5, melanoma differentiation-associated protein-5; MPO, myeloperoxidase; Neg, negative; NXP2, nuclear matrix protein 2; OJ, isoleucine; PM-Scl, PL-7, threonine; PL-12, alanine, polymyositis-scleroderma; PR3, proteinase 3; SAE1, small ubiquitin-like modifier activating enzyme; SRP, signal recognition particle; TIF1, transcriptional intermediary factor 1.

**Figure 2:**
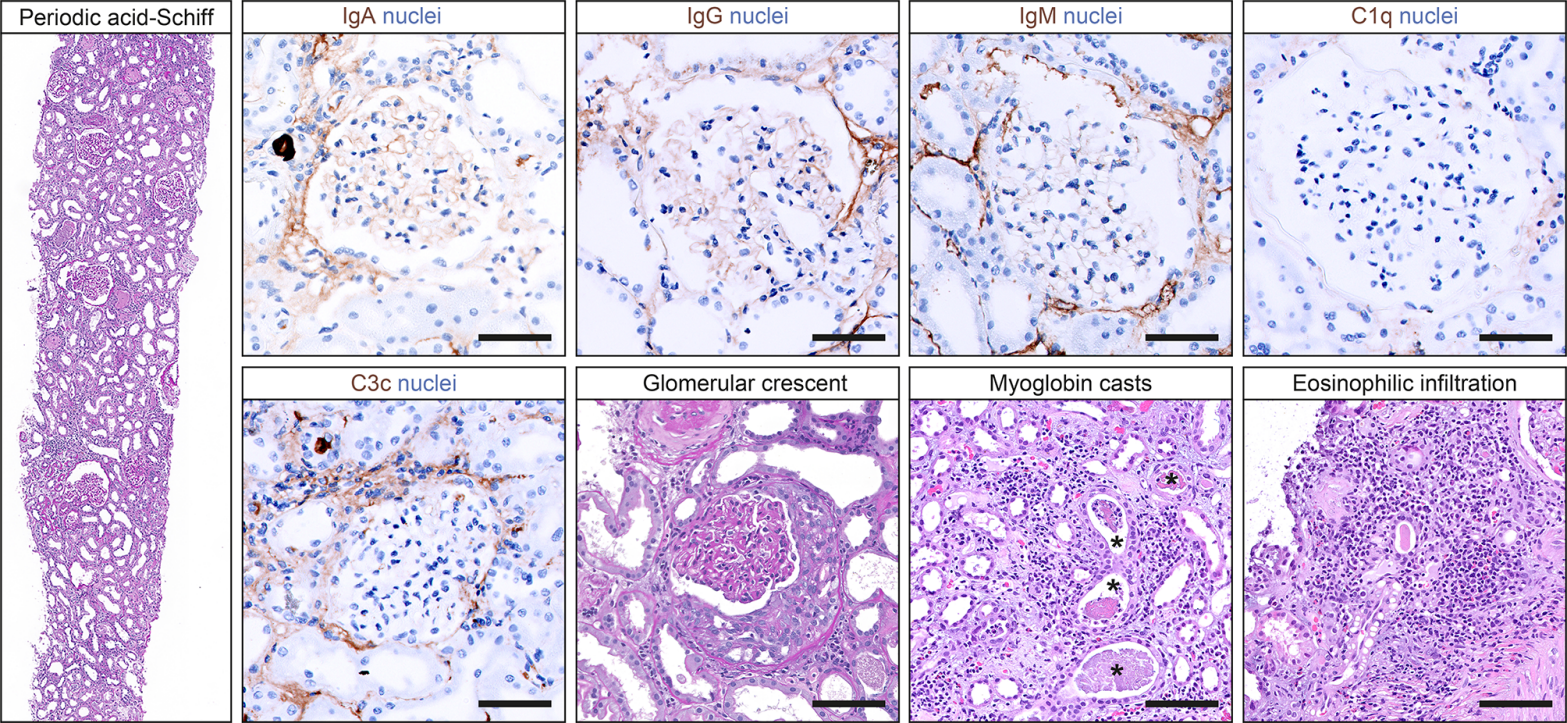
Histopathological findings in a kidney biopsy confirming pauci-immune crescentic GN. Representative photomicrographs of the kidney biopsy including staining for IgA (scale bar: 50 μm), IgG (scale bar: 50 μm), IgM (scale bar: 50 μm), C1q (scale bar: 50 μm), and C3c (scale bar: 50 μm); periodic acid-Schiff staining showing a glomerulus with crescent formation (scale bar: 50 μm); and hematoxylin/eosin staining with myoglobin casts (asterisks, scale bar: 100 μm) and tubulointerstitial inflammation with prominent eosinophilic infiltration (scale bar: 100 μm). C1q, complement component 1q; C3c, complement factor 3 conversion product; IgA, immunoglobulin A; IgG, immunoglobulin G; IgM, immunoglobulin M; GN, glomerulonephritis.

Based on suspected MPO-positive AAV, the patient received a steroid pulse with intravenous methylprednisolone for 3 days (250 mg per day) and oral prednisone 1 mg/kg daily thereafter (60 mg per day, **Figure 1D**). A kidney biopsy confirmed severe acute tubular injury with pauci-immune crescentic GN and interstital nephritis: cellular crescents in 1/15 (6.7%) glomeruli, global glomerular sclerosis in 2/15 (13.3%), mild (5%) interstitial fibrosis and tubular atrophy (IF/TA), interstitial inflammation (25%) with prominent eosinophilic infiltration, and severe acute tubular injury with myoglobin casts (**Figure 2** and **Table 2**). According to histopathological scoring, focal class ANCA GN and intermediate risk ANCA renal risk score (ARRS) were present (**Table 2**) (11, 12). Based on the diagnosis of AAV presenting with massive rhabdomyolysis and pauci-immune crescentic GN, intravenous cyclophosphamide (CYC) was initiated at 10 mg/kg (per CYCLOPS trial dosing, **Figure 1D**) (13). Thereafter, kidney function normalized without requirement of dialysis and proteinuria decreased to 1,603 mg/g creatinine and albuminuria to 351 mg/g creatinine (reference range: <30 mg/g, **Figures 1B, C**). Repeat serological testing confirmed that ANCA IF turned negative. Thereafter, oral prednisone was tapered down (currently 50 mg per day), and we do not plan to repeat administration of intravenous cyclophosphamide because rhabdomyolysis ceased and kidney function normalized.

**Table 2:** Histopathological findings in the kidney biopsy.

	Value
Lesions	
Total glomeruli—no.	15
Cellular crescents—no. (%)	1 (6.7)
Fibrocellular crescents—no. (%)	0 (0)
Global glomerular sclerosis—no. (%)	2 (13.3)
IF/TA—%	5
Interstitial inflammation—%	25
Scoring	
Berden class	Focal
ARRS	Intermediate risk

ARRS, ANCA renal risk score; IF/TA, interstitial fibrosis/tubular atrophy; no., number.

## Discussion

To our knowledge, we here present the first case of AAV presenting with massive rhabdomyolysis and pauci-immune crescentic GN after Pfizer-BioNTech COVID-19 mRNA vaccination. With millions of people being vaccinated for COVID-19, rare reports of adverse events are emerging. In this case, the temporal association between Pfizer-BioNTech COVID-19 mRNA vaccination and AAV presenting with rhabdomyolysis and pauci-immune crescentic GN suggests a neutrophilic immune response to mRNA as a potential trigger. This patient initially presented with upper thigh pain due to massive rhabdomyolysis after Pfizer-BioNTech COVID-19 mRNA vaccination. Rhabdomyolysis has been described in the context of COVID-19, and a direct viral tropism to myocytes has been suggested (14, 15). However, detection of SARS-CoV-2 infection in skeletal muscle cells has not been established yet (16). Rhabdomyolysis secondary to vaccination has previously been reported, mostly in the context of influenza vaccination (17, 18). In association with COVID-19 mRNA vaccination, the onset of fatigue, myalgias, and arthralgias following mRNA vaccination has been reported in a considerable subset of patients (19). Additionally, there is evidence that COVID-19 mRNA vaccination can directly induce myositis at the injection site, as previously observed in the deltoid muscle (20). In addition to rhabdomyolysis, we observed pauci-immune crescentic GN accompanied by MPO-ANCA autoantibodies after COVID-19 mRNA vaccination. To date, five cases of pauci-immune crescentic ANCA GN after the second dose of COVID-19 mRNA vaccination in all cases have been reported (7–10). In our case, kidney biopsy showed myoglobin casts due to massive rhabdomyolysis and pauci-immune crescentic GN, and it is likely that both contributed to deterioration of kidney function. It has already been reported that the first COVID-19 mRNA vaccination primes the innate immune system to mount a more potent response after the second booster immunization (21). It is possible that the enhanced immune response especially observed after the second dose of COVID-19 mRNA vaccination could be responsible for triggering the observed MPO-ANCA autoantibodies. Causal links between immune system activation by viral infections and AAV have been suggested due to onset of AAV predominantly during the winter (22, 23). Toll-like receptors (TLRs) are expressed on leukocytes and play crucial roles in the recognition of viral antigens, facilitating immune system activation and inflammation. Predominant TLR-2 and TLR-9 activation can stimulate autoimmunity in AAV, previously been described in the context of MPO-ANCA autoantibodies (24). Interestingly, TLR-2 activation in immunodominant cytotoxic T lymphocytes in response to SARS-CoV-2 S glycoprotein (as also produced by COVID-19 vaccines) has already been described (25). With regard to vaccination, there is some discussion about the relationship between vaccination and AAV recurrence in patients with pre-existing autoimmune disease after influenza vaccination as very rare but significant side effects (26). The temporal relationship could be explained theoretically, including molecular mimicry, polyclonal activation, or transient systemic proinflammatory cytokine responses that potentially provoke autoimmune diseases in genetically predisposed individuals (27). Interestingly, increased production of ANCA autoantibodies has already been described in response to viral mRNA-based influenza and rabies vaccines (27). Moreover, AAV and autoimmune reactions have been reported in the context of COVID-19, implicating a direct reaction to viral RNA (28–30). Therefore, the occurrence of AAV in the context of COVID-19 mRNA as compared with non-mRNA vaccines would be of great relevance. Huge vaccination programs are ongoing worldwide, and post-marketing surveillance systems must continue to assess vaccine safety important for the detection of any events associated with COVID-19 vaccination. This is especially relevant in complex diseases where diagnosis is often challenging, as in our patient with AAV presenting with massive rhabdomyolysis and pauci-immune crescentic GN. The limitation of this case report is only a temporal relationship between COVID-19 mRNA vaccination and onset of AAV. However, AAV onset in association with COVID-19 mRNA vaccination has independently been observed before and requires further investigation with regard to the mechanisms linking autoimmunity to COVID-19 vaccines (7–9). Fortunately, treatment of AAV is possible and caution in such cases is warranted with regard to early testing if clinical symptoms are compatible with AAV in principle.

## Data Availability Statement

The original contributions presented in the study are included in the article/supplementary material. Further inquiries can be directed to the corresponding author.

## Ethics Statement

Ethical review and approval was not required for the study on human participants in accordance with the local legislation and institutional requirements. The patients/participants provided their written informed consent to participate in this study.

## Author Contributions

BT was directly involved in the treatment of the patient, conceived the case report, collected and analyzed the data, and wrote the manuscript. SH evaluated kidney biopsy findings and edited the manuscript. All authors contributed to the article and approved the submitted version.

## Funding

We acknowledge the support from the Open Access Publication Funds of the Georg August University Göttingen.

## Conflict of Interest

The authors declare that the research was conducted in the absence of any commercial or financial relationships that could be construed as a potential conflict of interest.

## Publisher’s Note

All claims expressed in this article are solely those of the authors and do not necessarily represent those of their affiliated organizations, or those of the publisher, the editors and the reviewers. Any product that may be evaluated in this article, or claim that may be made by its manufacturer, is not guaranteed or endorsed by the publisher.
